# Different responses of ecosystem carbon exchange to warming in three types of alpine grassland on the central Qinghai–Tibetan Plateau

**DOI:** 10.1002/ece3.3741

**Published:** 2017-12-30

**Authors:** Hasbagan Ganjurjav, Guozheng Hu, Yunfan Wan, Yue Li, Luobu Danjiu, Qingzhu Gao

**Affiliations:** ^1^ Institute of Environment and Sustainable Development in Agriculture Chinese Academy of Agricultural Sciences Beijing China; ^2^ Key Laboratory for Agro‐Environment Ministry of Agriculture Beijing China; ^3^ Nagqu Grassland Station Tibet Autonomous Region Nagqu China

**Keywords:** alpine grasslands, carbon exchange, Qinghai–Tibetan Plateau, warming

## Abstract

Climate is a driver of terrestrial ecosystem carbon exchange, which is an important product of ecosystem function. The Qinghai–Tibetan Plateau has recently been subjected to a marked increase in temperature as a consequence of global warming. To explore the effects of warming on carbon exchange in grassland ecosystems, we conducted a whole‐year warming experiment between 2012 and 2014 using open‐top chambers placed in an alpine meadow, an alpine steppe, and a cultivated grassland on the central Qinghai–Tibetan Plateau. We measured the gross primary productivity, net ecosystem CO_2_ exchange (NEE), ecosystem respiration, and soil respiration using a chamber‐based method during the growing season. The results show that after 3 years of warming, there was significant stimulation of carbon assimilation and emission in the alpine meadow, but both these processes declined in the alpine steppe and the cultivated grassland. Under warming conditions, the soil water content was more important in stimulating ecosystem carbon exchange in the meadow and cultivated grassland than was soil temperature. In the steppe, the soil temperature was negatively correlated with ecosystem carbon exchange. We found that the ambient soil water content was significantly correlated with the magnitude of warming‐induced change in NEE. Under high soil moisture condition, warming has a significant positive effect on NEE, while it has a negative effect under low soil moisture condition. Our results highlight that the NEE in steppe and cultivated grassland have negative responses to warming; after reclamation, the natural meadow would subject to loose more C in warmer condition. Therefore, under future warmer condition, the overextension of cultivated grassland should be avoided and scientific planning of cultivated grassland should be achieved.

## 1 INTRODUCTION

Since the 19th century, the global climate has undergone continuous warming, and this is driving changes in ecosystem structure and function (IPCC, 2014). Carbon exchange in terrestrial ecosystems is a major regulator of the CO_2_ balance between vegetation and the atmosphere and is an important part of the global carbon cycle (Beer et al., 2010; Mahecha et al., 2010). Climate factors, including temperature and precipitation, have significant effects on ecosystem CO_2_ exchange, and changes in ecosystem C exchange also have a feedback effect on the climate (Luo, Wan, Hui, & Wallace, 2001). Ecosystem C exchange includes gross ecosystem primary productivity (GEP; representing carbon uptake) and ecosystem respiration (ER; representing carbon emission). The net carbon uptake of the ecosystem is represented by net ecosystem CO_2_ exchange (NEE), which is the difference between GEP and ER (Trumbore, 2006). In terrestrial ecosystems, ER comprises soil respiration (SR) and above‐ground respiration (Trumbore, 2006).

In grasslands, temperature and precipitation are the major factors influencing carbon uptake. A meta‐analysis of grassland ecosystems worldwide has shown that approximately 69% and 37% of GEP are controlled by precipitation and temperature, respectively (Beer et al., 2010). However, in some cold regions, the effect of temperature may be greater than that of precipitation (Beer et al., 2010). Warming usually has a positive effect on GEP in grasslands, especially in cold regions (Sekine, 2013). However, in a semiarid grassland, warming has been shown to have a negative effect on GEP (Li et al., 2017). Fu et al. (2013) found that experimental warming had no significant effect on gross primary production in an alpine meadow on the Qinghai–Tibetan Plateau. Therefore, change in water availability in grassland ecosystems may be an important factor influencing the GEP response to increasing temperature.

Ecosystem C emissions are derived from soil respiration and above‐ground respiration. Both these parameters typically increase with warming, resulting in enhanced ER (Wu, Dijkstra, Koch, Penuelas, & Hungate, 2011). However, in some situations, respiration can decline under warming conditions, as plants respond to the effects of heat and drought (Li et al., 2017). The sensitivity of respiration to temperature is an indicator of the response of ER to warming, but previous studies reported that the sensitivity is not consistent across terrestrial ecosystems (Peng, Piao, Wang, Sun, & Shen, 2009; Zhou, Shi, Hui, & Luo, 2009). Consequently, ER responds differently to warming depending on the biome involved.

Grassland ecosystems are generally considered to be carbon sinks, although spatiotemporal variations occur in NEE among grasslands (Borchard et al., 2015). The effects of warming on the NEE of a grassland ecosystem depend on the response of GEP and ER to warming. Variations in NEE are driven mainly by water availability, ecosystem structure, and temperature. Therefore, under warming conditions, the response of NEE is ecosystem‐dependent. In dry grasslands, warming increases net C uptake during rainy periods but decreases net C uptake in dry periods (Grant, Baldocchi, & Ma, 2012). However, a recent meta‐analysis of terrestrial ecosystems worldwide showed that warming does not affect NEE significantly (Wu et al., 2011). Taken together, the response of NEE to warming in grasslands is very complicated.

Ecosystem C exchange is regulated mainly by temperature and precipitation (Beer et al., 2010), but the vegetation community structure and land management practices are also important factors (Abdalla et al., 2013; Cable et al., 2012). Therefore, in different types of ecosystem having different temperatures and water availability and utilization, warming may have different effects on ecosystem C exchange. The combined effects of temperature, water conditions, land use type, and ecosystem type tend to increase the uncertainties associated with any evaluation of how ecosystem C exchange responds to warming.

The Qinghai–Tibetan Plateau in southwest China is referred to as the “third pole” of the world because of its high elevation and low temperatures (Qiu, 2008). Alpine grassland is the major ecosystem type on the plateau, covering ~50% of the area (Ni, 2000). Areas of alpine meadow and alpine steppe cover ~10% and ~30% of the plateau, respectively (Ni, 2000). The alpine grasslands support grazing by yak, Tibetan sheep, and endangered ungulates. To address the forage shortage for domestic grazing and to restore degraded grasslands, some areas of the plateau have been planted with forage crops (Dong et al., 2010). The grassland used for forage is different from the native grasslands in terms of productivity and ecosystem C exchange. The alpine grasslands, which include alpine meadows, alpine steppes, and cultivated grasslands, are important for the stability of the ecosystem and its functioning and also support both herders and grazers on the plateau. These three types of grassland are subjected to similar temperatures, but there are marked differences in precipitation between the meadow and steppe regions, and the soil water content in the cultivated grassland is lower than in the natural grasslands.

The Qinghai–Tibetan Plateau has been subjected to significant warming since the 1960s, and the rate of warming in this area has been higher than in other parts of China (Chen et al., 2013; Piao et al., 2004). Moreover, the total annual precipitation in this region has increased slightly in the past 50 years (Chen et al., 2013), but the level of precipitation in the growing season has decreased slightly (Li, Yang, Wang, Zhu, & Tang, 2010). These changes, combined with the increased evapotranspiration resulting from warming, suggest that the plateau will become warmer and dryer in the future, especially during the growing season (Gao, Li, Xu, Wan, & Jiangcun, 2014). The high evaporation rate on the plateau differs from that on alpine tundra, which is also a cold region at high elevations. The alpine grassland is dominated by herbs, while alpine tundra is covered by evergreen species, mosses, and lichens. Therefore, the mechanisms by which alpine grasslands on the Qinghai–Tibetan Plateau respond to warming differ from the mechanisms used by tundra species. Studies investigating warming impacts on the plateau have shown a positive effect on ecosystem C exchange in alpine meadow regions (Peng et al., 2014; Ganjurjav et al., 2015; Chen et al., 2017). However, our previous studies showed that drought in the growing season resulted in a periodic decrease in NEE under warming conditions in meadow regions (Ganjurjav, Gao, Schwartz et al., 2016). Although many studies have examined the response of this type of grassland to warming on the plateau, no warming experiments have comprehensively considered the different grassland types. It is also unknown how ecosystem C exchange is regulated under warming conditions in the different types of alpine grassland, or how exchange is affected by different water availability conditions in this high and cold region.

In this study, we used open‐top chambers (OTCs) placed in alpine meadow, alpine steppe, and cultivated grassland areas on the central Qinghai–Tibetan Plateau to investigate the effects of increased temperature on the different types of grassland. We measured the ecosystem C exchange, including GEP, NEE, ER, and SR, using a static chamber method. Based on the findings of previous studies, we hypothesized that warming would have different effects on NEE in meadow, steppe, and cultivated grasslands, mainly because of the different water conditions among these systems.

## 2 MATERIALS AND METHODS

### 2.1 Site descriptions

**Figure 1:**
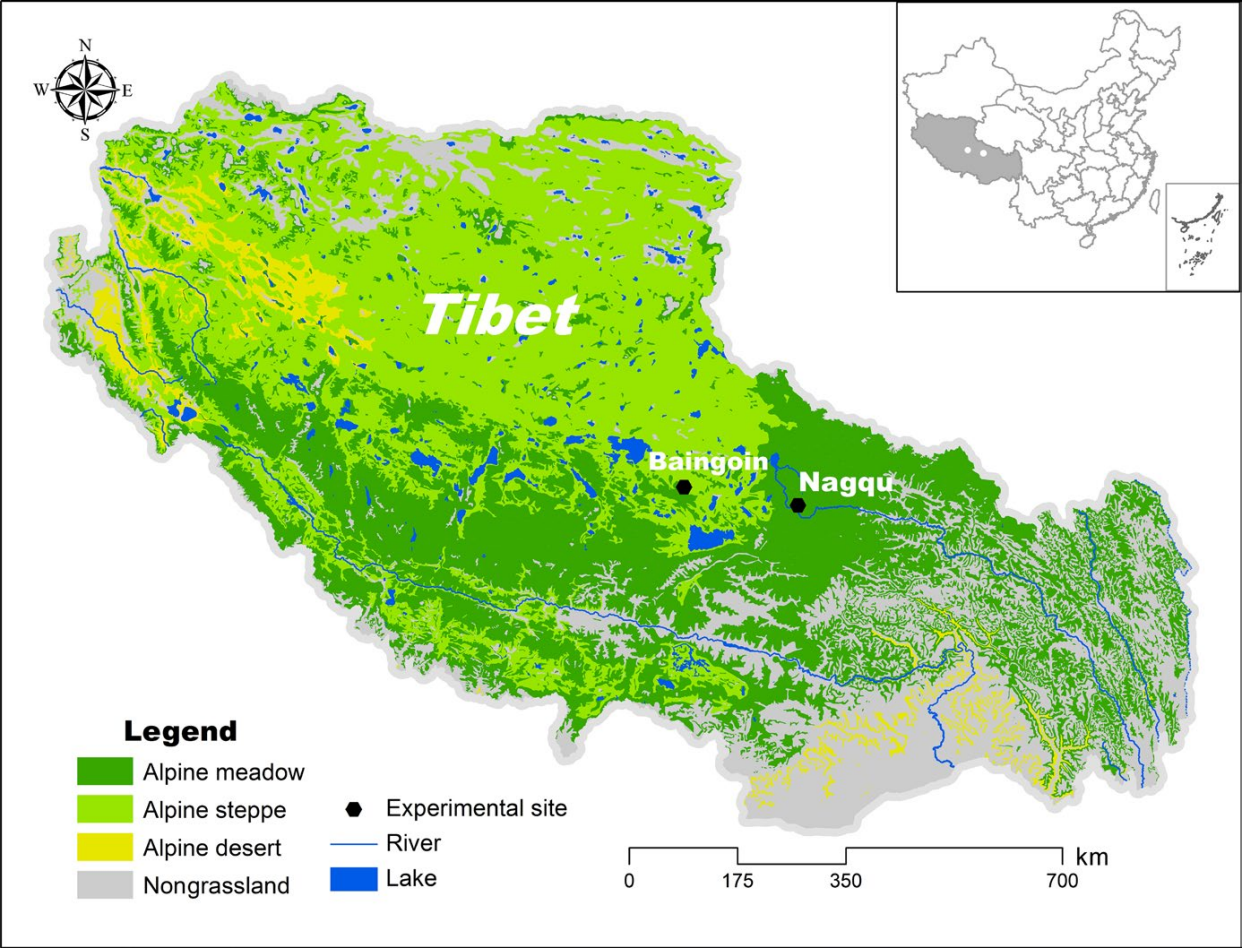
Locations of experimental area

**Figure 2:**
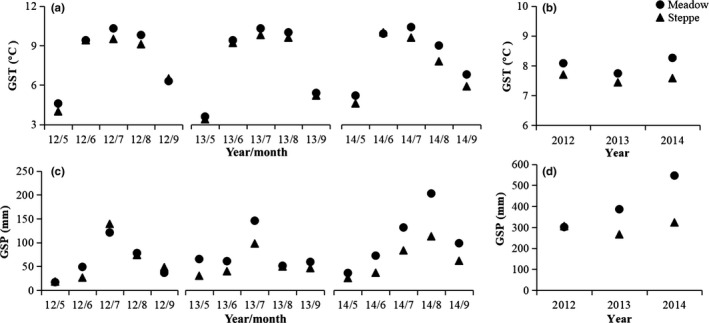
Growing season temperature (GST) and growing season precipitation (GSP) in Nagqu (meadow and cultivated grassland) and Bangion (steppe) from 2012 to 2014. (a) Seasonal variation in growing season temperature; (b) annual variation in growing season temperature; (c) seasonal variation in growing season precipitation; (d) annual variation in growing season precipitation

The experimental sites were located in the Nagqu (31.441°N, 92.017°E; 4,460 m above sea level) and Baingoin (31.389°N, 90.028°E; 4,725 m above sea level) counties, Nagqu Prefecture, Tibet Autonomous Region, China (Figure 1). The sites located in Nagqu County are characterized by alpine meadow and cultivated grassland habitats. The site in Baingoin County is characterized by an alpine steppe habitat. During the experimental period (2012–2014), the mean growing season temperatures in Nagqu County and Baingoin County are 8.0°C and 7.6°C, respectively, and the total growing season precipitation is 411.2 mm and 298.3 mm, respectively (Figure 2). The dominant plant species are *Carex moorcroftii*,* Kobresia pygmaea*,* Lancea tibetica*,* Poa pratensis*, and *Potentilla acaulis* at the meadow site, and *Festuca ovina*,* Koeleria argentea*,* Leontopodium nanum*,* Oxytropis microphylla*, and *Stipa purpurea* at the steppe site. The details for community differences between meadow and steppe habitat are provided in Ganjurjav, Gao, Gornish et al. (2016). The cultivated grassland is dominated by *Elymus nutans*, which was planted in 2010 at alpine meadow region. Warming induced ANPP increase in meadow and cultivated grassland while decrease ANPP in steppe (Table S1, Ganjurjav, Gao, Gornish et al., 2016). The soil organic carbon and total nitrogen were increased in meadow under warming compared to control while decreased in steppe and cultivated grassland under warming compared to control (Table S1, Ganjurjav, Gao, Gornish et al., 2016). Before the experiment, yaks grazed the experimental sites. However, the sites were fenced in 2010 and were not grazed or mown during the experimental period.

### 2.2 Experimental design

The OTCs were used to simulate whole‐year warming in our experiment. They were made of solar radiation‐transmitting plastic and had the dimensions: height, 0.45 m; diameter at ground height, 1.20 m; and diameter at maximum height, 0.65 m. We began the warming experiment in July 2011, with an experimental design involving four replicates of each of the control and the warming treatment (randomly distributed), giving a total of eight plots at each of the meadow, steppe, and cultivated grassland sites. The area of each experimental site was 2,500 m^2^.

### 2.3 Microclimates measurements

We used the EM50 Data Collection System (Decagon Devices, Inc., NE, USA) for microclimate measurements. We measured the soil temperature and water content at 5‐cm depth at 30‐min intervals in each plot and site throughout the growing season (early June to late August). There have no microclimates data during the nongrowing season.

### 2.4 Ecosystem C exchange measurements

A chamber‐based method was used to measure ecosystem fluxes. We used a portable photosynthesis system (Li‐6400; LI‐COR Inc., Lincoln, NE, USA) and the transparent static chamber method to measure NEE and ER in each plot during the growing seasons from 2012 to 2014. Before the experiment, a plastic base (30 × 30 cm^2^) was embedded (3‐cm depth) in each plot area to facilitate measurements. We measured NEE three times per month between 10:00 and 12:00 on sunny days from June to August in 2012 and 2013 and from May to September in 2014. To make these measurements, we placed a transparent polyethylene chamber (30 cm × 30 cm × 40 cm) on the base of each plot, placed a fan on the roof of the chamber to mix the gases inside, and measured NEE over a period of 90s. After the measurement was completed, we removed the chamber and allowed the air humidity and CO_2_ values to reach ambient levels. We then replaced the chamber on the base, covered it with shade cloth, and measured ER over a period of 90s. We calculated GEP as the sum of the NEE and ER values. In this study, the positive value of NEE indicates carbon uptake while negative one indicates carbon loss from ecosystem. We used a portable flux meter (West Systems, Italy) and chamber method to determine SR over a period of 90s in each plot.

### 2.5 Data analysis

The seasonal mean values of measurement data were calculated from the monthly mean values for June, July, and August, which were the averages of all the measurements obtained in a given month. We used the IBM SPSS version 20.0 statistical package for the data analysis. *T* test was used to examine the C flux values difference between warming and control to explore the effect of warming. Repeated measures analysis of variance (RMANOVA) was used to investigate the effects of warming, year, site, and their interactions on the mean seasonal ecosystem C fluxes. RMANOVA was also used to investigate the effects of warming on the ecosystem C flux in each growing season for the period 2012**–**2014. The seasonal mean data for ecosystem C flux, soil temperature, and soil water content were used to run a stepwise multiple linear regression analysis to investigate the correlation of ecosystem C flux to soil temperature and soil water content.

## 3 RESULTS

### 3.1 Microclimates

**Figure 3:**
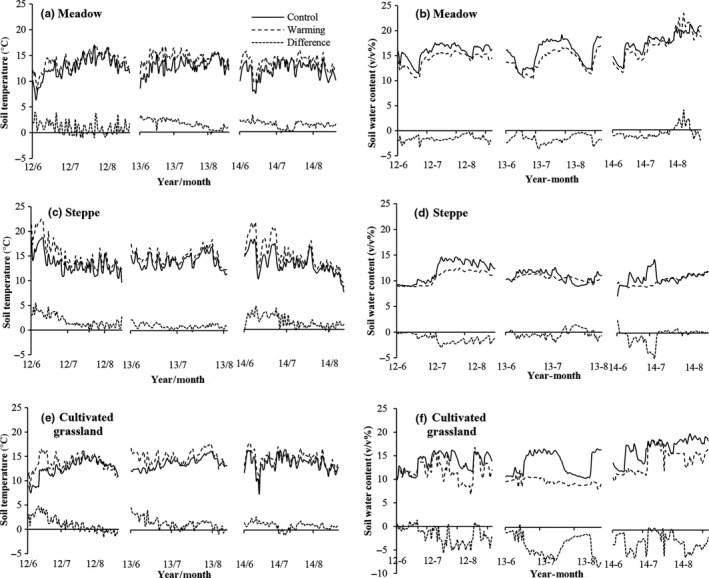
Average soil temperature (a, b, c), soil water content (d, e, f), and their differences between warming and control plots in each treatment in alpine meadow, alpine steppe, and cultivated grassland in growing season (June to August) from 2012 to 2014

Warming had a significant effect on the temperature of the shallow soil layer in the alpine meadow, alpine steppe, and cultivated grassland during the growing seasons from 2012 to 2014 (Figure 3a–c). At the meadow site, the soil temperature increased by 1.4°C in the warming treatment plots compared with the control (*p *<* *.05, Figure 3a). At the steppe site, the mean growing season soil temperatures were 13.5°C and 15.0°C in the control and warming plots, respectively (Figure 3b). In the cultivated grassland, the temperatures reached 13.3°C and 14.4°C in the control and warming plots, respectively (Figure 3c). Warming increased the soil temperature by 1.5°C and 1.1°C in the steppe and cultivated grassland, respectively (*p *<* *.05, Figure 3b,c).

In contrast to the soil temperature, the soil water content was significantly lower in the treatment plots than in the control plots at all three sites (Figure 3d–f). The mean growing season soil water content was 15.6%, 11.2%, and 14.6% in the control plots at the alpine meadow, alpine steppe, and cultivated grassland sites, respectively. In the warming treatment plots, the mean soil water content in the alpine meadow, alpine steppe, and cultivated grassland sites was lower than in the controls by 1.2% (14.4%), 0.7% (10.5%), and 2.7% (12.0%), respectively (*p *<* *.05, Figure 3d–f).

### 3.2 Spatiotemporal patterns of ecosystem C flux

**Figure 4:**
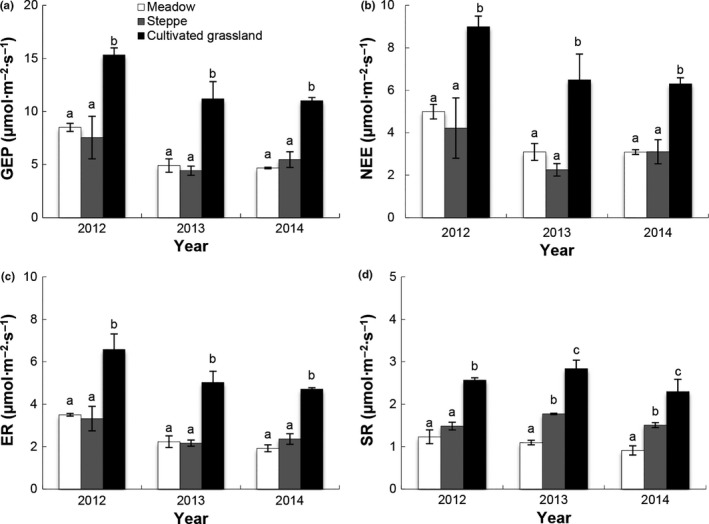
Growing seasonal mean (±*SE*) (a) gross ecosystem productivity (GEP), (b) net ecosystem CO_2_ exchange (NEE), (c) ecosystem respiration (ER), and (d) soil respiration (SR) in control plots in meadow, steppe, and cultivated grassland from 2012 to 2014. Different letters on the bar indicated that significant differences among each grassland type in the same year

The ecosystem C fluxes differed among the alpine meadow, alpine steppe, and cultivated grassland areas on the plateau (*p *<* *.05, Table 1, Figure 4). There were no significant differences in GEP, NEE, or ER between the alpine meadow and alpine steppe sites from 2012 to 2014 (*p *>* *.05). The SR was significantly higher at the alpine steppe site than at the alpine meadow site in 2013 and 2014 (*p *<* *.05). The values for GEP, NEE, ER, and SR were significantly higher at the cultivated grassland site than at the alpine meadow or alpine steppe sites (*p *<* *.05, Figure 4). The GEP, NEE, ER, and SR values for the cultivated grassland were 80.4%, 80.1%, 87.8%, and 108.3% higher than those for the alpine meadow, respectively (*p *<* *.05).

**Table 1:** Results of repeated measures analysis of variance (RMANOVA) on the effects of year (*Y*), warming (*W*), grassland type (*T*) and their interaction on gross ecosystem productivity (GEP), net ecosystem CO_2_ exchange (NEE), ecosystem respiration (ER), and soil respiration (SR) in central Qinghai–Tibetan Plateau

Factors	Ecosystem C fluxes											
	GEP			NEE			ER			SR		
	Mean square	*F* value	*p* Value	Mean square	*F* value	*p* Value	Mean square	*F* value	*p* Value	Mean square	*F* value	*p* Value
*Y*	221.6	134.5	**<.001**	78.4	65.5	**<.001**	31.2	130.9	**<.001**	0.81	5.26	**.007**
*W*	9.65	5.86	**.001**	6.90	5.71	**.001**	1.45	6.09	**.001**	1.88	12.3	**<.001**
*T*	437.5	265.6	**<.001**	133.5	111.5	**<.001**	104.4	438.4	**<.001**	38.2	250.0	**<.001**
*Y* × *W*	2.34	1.42	.217	1.07	0.89	.504	0.37	1.50	.190	0.43	0.28	.945
*Y* × *T*	7.28	4.42	**.003**	3.39	2.83	**.029**	0.72	3.04	**.022**	0.78	5.11	**.001**
*W* × *T*	8.90	5.40	**<.001**	6.65	5.56	**<.001**	1.35	5.66	**<.001**	1.09	7.14	**<.001**
*Y* × *W* × *T*	1.39	0.84	.608	0.76	0.63	.808	0.31	1.26	.257	0.13	0.85	.596

The bold values indicate significant effect at the p < 0.05 level.

### 3.3 Annual variations in ecosystem C flux under warming

**Figure 5:**
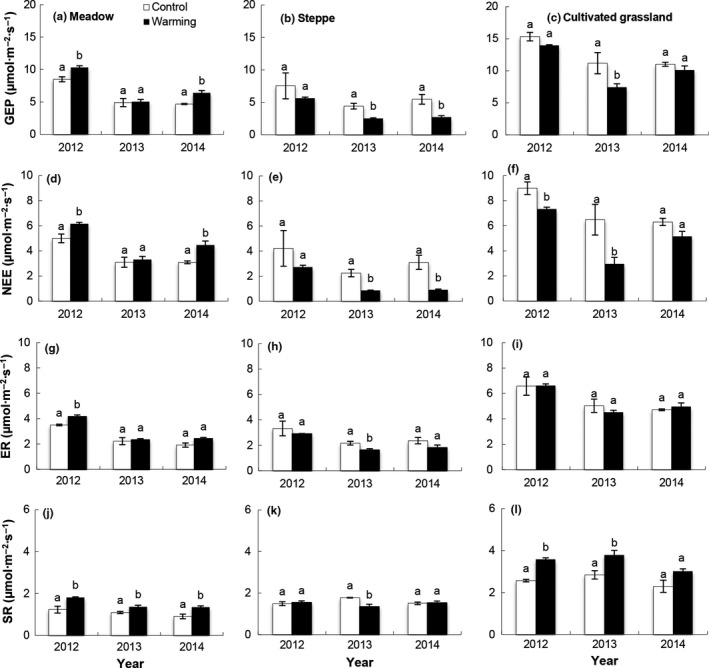
Growing seasonal mean (±*SE*) ecosystem C fluxes in control and treatment plots in (a, d, g, j) meadow, (b, e, h, k) steppe, and (c, f, i, l) cultivated grassland from 2012 to 2014. Different letters on the bar indicated that significant differences between control and warming in the same year

Warming and grassland type had significant effects on the growing season ecosystem C exchange in the alpine grasslands of the plateau (Table 1). At the alpine meadow site, warming significantly increased the GEP, NEE, ER, and SR in 2012 and 2014, but not ER in 2014 (*p *<* *.05). In 2013, there was no significant difference in the levels of GEP, NEE, or ER between the warming treatment and control sites (*p *>* *.05, Figure 5). On the alpine steppe and cultivated grassland, warming generally reduced the ecosystem C exchange, but not SR in the cultivated grassland (Figure 5).

### 3.4 Seasonal variations in ecosystem C flux under warming

**Figure 6:**
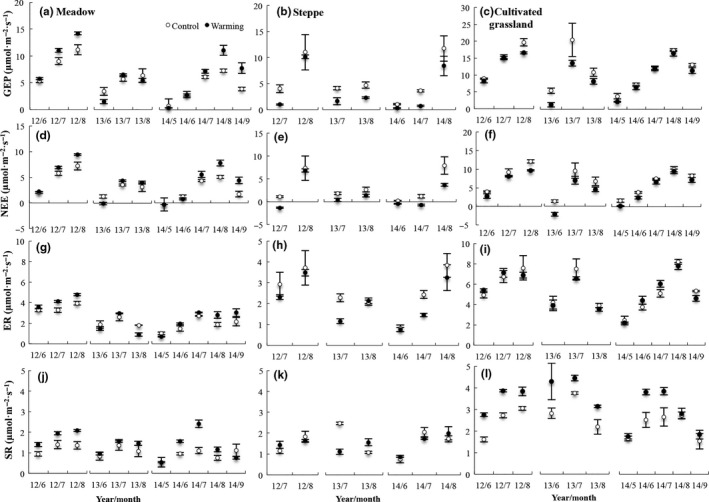
Seasonal variation in ecosystem C fluxes (±*SE*) in control and treatment plots in (a, d, g, j) meadow, (b, e, h, k) steppe, and (c, f, i, l) cultivated grassland from 2012 to 2014

There were significant seasonal variations in ecosystem C exchange at the alpine meadow, alpine steppe, and cultivated grassland sites from 2012 to 2014 (*p *<* *.001, Table 2, Figure 6). At the alpine meadow site, warming significantly changed the seasonal patterns of GEP and NEE during the experimental period (*p *<* *.05) but had not significantly changed the ER and SR, except in 2012 (Table 2, Figure 6). At the alpine steppe site, the seasonal patterns of GEP, ER, and SR were significantly affected by warming in 2013 (*p *<* *.05). At the cultivated grassland site, seasonal patterns of GEP, NEE, and SR were significantly affected by warming in 2012 and 2013 (Table 2, Figure 6). These results suggest that warming influenced the level of ecosystem C exchange and its seasonal patterns. However, the effects were not consistent among the three sites.

**Table 2:** Results (*p* values) of repeated measures analysis of variance (RMANOVA) on the effects of warming (*W*), measuring date (*D*) and their interaction on gross ecosystem productivity (GEP), net ecosystem CO_2_ exchange (NEE), ecosystem respiration (ER), and soil respiration (SR) in central Qinghai–Tibetan Plateau from 2012 to 2014

Ecosystem C fluxes	Year	Grassland types								
		Meadow			Steppe			Cultivated grassland		
		*W*	*D*	*W* × *D*	*W*	*D*	*W* × *D*	*W*	*D*	*W* × *D*
GEP	2012	**0.012**	**<0.001**	**<0.001**	0.354	**<0.001**	0.545	**0.019**	**<0.001**	**0.015**
	2013	0.768	**<0.001**	**<0.001**	**0.006**	**<0.001**	**0.019**	0.087	**<0.001**	**0.015**
	2014	**0.025**	**<0.001**	**0.011**	**0.024**	**<0.001**	0.554	0.65	**<0.001**	0.6
NEE	2012	**0.009**	**<0.001**	**<0.001**	0.469	**<0.001**	0.601	0.114	**<0.001**	**0.035**
	2013	0.942	**<0.001**	**<0.001**	**0.026**	**0.003**	0.147	0.137	**<0.001**	**0.007**
	2014	**0.037**	**<0.001**	**0.012**	**0.02**	**<0.001**	0.570	0.225	**<0.001**	0.657
ER	2012	**0.022**	**<0.001**	**0.002**	**0.037**	**<0.001**	0.187	0.061	**<0.001**	0.195
	2013	0.4	**<0.001**	0.39	0.097	**<0.001**	**0.002**	0.215	**<0.001**	0.105
	2014	0.191	**<0.001**	0.362	**0.04**	**<0.001**	0.645	0.532	**<0.001**	0.206
SR	2012	**0.006**	**<0.001**	0.124	0.062	**0.04**	0.212	**0.02**	**<0.001**	**0.017**
	2013	0.648	**<0.001**	0.482	0.523	0.475	**0.035**	0.248	**<0.001**	**0.035**
	2014	0.065	**<0.001**	0.103	0.095	**<0.001**	0.066	**0.013**	**<0.001**	0.188

**Figure 7:**
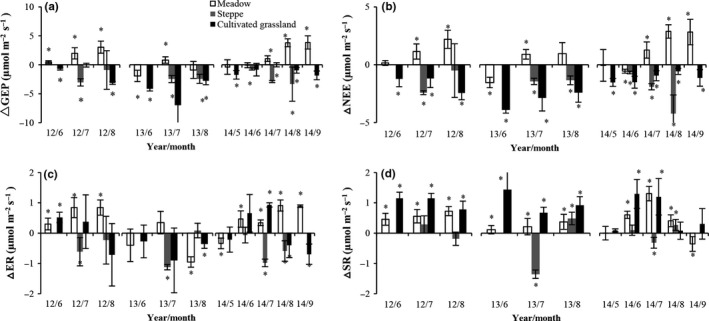
Seasonal variation in ecosystem carbon flux differences (±*SE*) between warming and control plots from 2012 to 2014 in meadow, steppe, and cultivated grassland. “*” on the bar indicated that the value significantly different from zero

The seasonal patterns of difference in ecosystem C flux between the warming and control plots are shown in Figure 7. At the meadow site, the GEP and NEE increased significantly in the warming plots compared with the control plots in July from 2012 to 2014 (*p *<* *.05), but decreased significantly in June 2013, and increased or did not change in June in 2012 and 2014. Consequently, in 2013, the mean values for GEP and NEE under warming conditions were not different from those under ambient conditions (Figure 5a,d). In the steppe, the GEP, NEE, ER, and SR values all decreased with time in the warming treatments compared with the control, except for SR in August 2013 and 2014 (Figure 7). In the cultivated grassland, the SR value increased significantly in the warming treatment compared with the control in all 3 years (*p *<* *.05, Figure 7).

### 3.5 Ecosystem C fluxes over 3 years of warming

**Figure 8:**
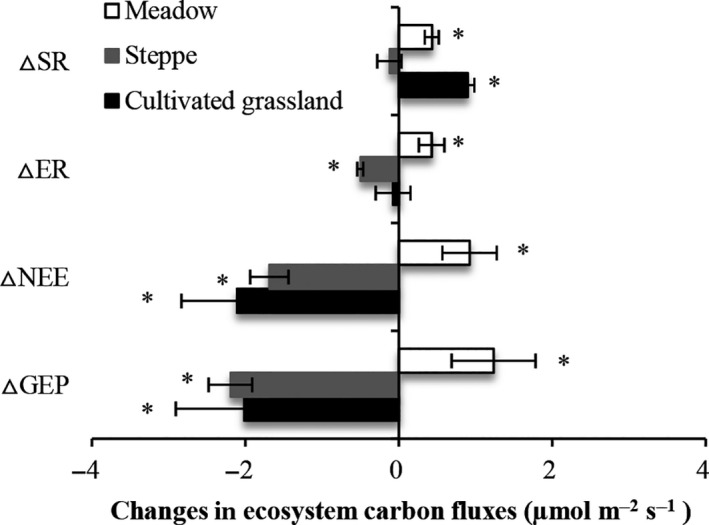
Three years’ (2012–2014) average changes in ecosystem C fluxes (±*SE*) under warming compared to control plot in meadow, steppe, and cultivated grassland. “*” on the bar indicated that the value significantly different from zero

We compared the mean ecosystem carbon flux over the 3 years of the study period between the warming treatment and control plots. At the alpine meadow site, the GEP, NEE, ER, and SR in the warming treatments increased by 20.4%, 25.0%, 16.7%, and 39.8%, respectively, relative to the controls (*p *<* *.05, Figure 8). At the alpine steppe site, relative to the control GEP, NEE, and ER values in the warming treatment decreased by 39.7%, 55.8%, and 20.2%, respectively (*p *<* *.05), but warming had no significant effect on SR (*p *>* *.05, Figure 8). At the cultivated grassland, the GEP and NEE decreased significantly under warming conditions compared with the controls (*p *<* *.05), whereas ER was not affected (*p *>* *.05), and the level of SR increased significantly (*p *<* *.05, Figure 8).

### 3.6 Effects of soil temperature and soil water content on ecosystem C exchange

**Figure 9:**
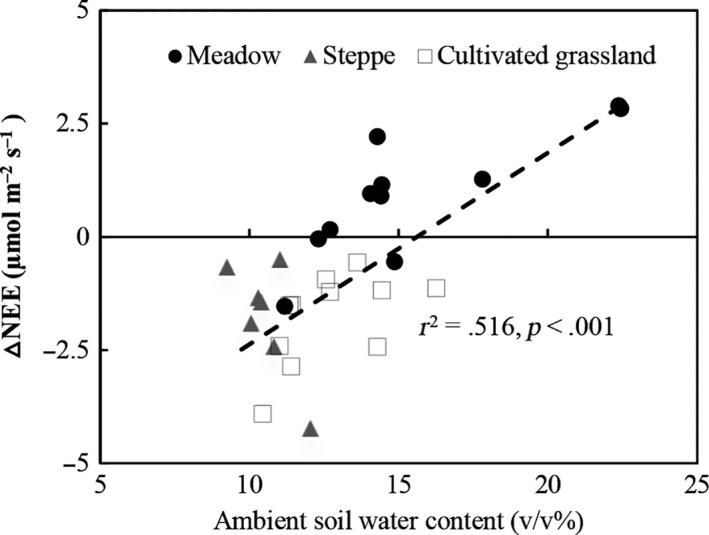
Correlations of NEE difference (ΔNEE) between warming and control plot and ambient soil water content in three types of alpine grassland on the central Qinghai–Tibetan Plateau across three experimental years (2012–2014)

Soil temperature and soil water content are important regulators of ecosystem C exchange in the alpine grasslands of the Qinghai–Tibetan Plateau (Table S2). However, they had differing effects depending on the treatment and grassland type. The standardized coefficients of stepwise regression analysis between ecosystem C exchange and soil temperature and soil water content are listed in Table 3. At the meadow site, the soil temperature and soil water content were positively correlated with ecosystem C exchange. The standardized coefficients for GEP, NEE, and ER in relation to soil temperature and soil water content were less different from each other in the control plot at the meadow site (Table 3). However, under warming conditions, the standardized coefficient for GEP and NEE in relation to soil water content (0.438 and 0.475) was higher than that with respect to soil temperature (0.305 and 0.301) at the meadow site. At the steppe site, the soil temperature was negatively correlated with ecosystem C exchange, and the standardized coefficients were greater in the warming treatment than in the control (Table 3). For the cultivated grassland, both soil temperature and water content contributed to change in the GEP and NEE in the control plots, but only the soil water content was a significant regulator under warming conditions (Table 3). The ambient soil water content was positively correlated (*p *<* *.001) with warming‐induced change in the NEE (ΔNEE) in the three grassland types across the 3 years of the experiment (Figure 9).

**Table 3:** Standardized coefficients of correlation model in ecosystem carbon fluxes (GEP, NEE, ER, and SR) to soil temperature (ST) and soil water content (SWC) in three types of alpine grassland

Ecosystem types	Treatments	Soil properties	Ecosystem carbon fluxes			
			GEP	NEE	ER	SR
Meadow	Control	ST	0.317	0.367	0.221	0.251
		SWC	0.307	0.326	0.217	—
	Warming	ST	0.305	0.301	0.364	0.370
		SWC	0.438	0.475	—	—
Steppe	Control	ST	−0.528	−0.448	−0.652	—
		SWC	—	—	—	—
	Warming	ST	−0.643	−0.525	−0.832	−0.719
		SWC	—	—	—	—
Cultivated grassland	Control	ST	0.331	0.287	0.358	—
		SWC	0.438	0.456	0.356	—
	Warming	ST	—	—	0.433	0.284
		SWC	0.427	0.469	0.283	—

## 4 DISCUSSION

Our experimental results provide evidence for the effects of warming on ecosystem C exchange in alpine meadows, alpine steppes, and cultivated grasslands of the central Qinghai–Tibetan Plateau. Consistent with our hypothesis, warming only increased the ecosystem C uptake in some years (2012 and 2014) in the alpine meadow and reduced the C uptake in the alpine steppe and cultivated grassland. We found that both soil temperature and soil water content were important regulators of ecosystem C exchange. In particular, the soil water content was significantly correlated with the difference in NEE between the warming treatments and the control. Our study highlights the important role of soil water content in dictating the response of ecosystem C exchange to warming in alpine grassland habitats on the Qinghai–Tibetan Plateau. Moreover, the reclamation of natural meadow would induce more carbon loss from grassland ecosystems.

### 4.1 Effects of temperature and moisture on C flux in alpine grasslands

Variations in ecosystem C exchange are usually driven by temperature, precipitation, soil heterogeneity, and the type of land management (Borchard et al., 2015; Elsgaard et al., 2012). Our results show that in the control plots in the meadow and cultivated grassland areas, both soil temperature and soil water content were important regulators and contributed equally to variations in ecosystem C exchange. However, under warming conditions, the soil water content became more important than temperature in determining ecosystem C uptake in the meadow and cultivated grassland habitats because of warming treatment reduced soil moisture content and even induced water shortage. Fu et al. (2009) compared the NEE of temperate steppes, alpine shrub‐meadows, and alpine meadow‐steppes and showed that the interannual and intersite variations in annual NEE were controlled mainly by the available soil moisture. At the alpine steppe site, the ecosystem C exchange was negatively correlated with soil temperature. At the alpine steppe, a low soil water content may resulted in negative effects of warming on ecosystem carbon uptake. Hence, under warming conditions in the alpine grasslands of the Qinghai–Tibetan Plateau, soil drying rather than temperature may be the main limiting factor for ecosystem C uptake under warming conditions (Ganjurjav et al., 2015).

### 4.2 Different responses of C flux to warming among three types of grassland

Alpine meadow ecosystems are characterized by high rates of photosynthesis and low respiration rates, because the levels of precipitation are high and the temperatures are low during the growing season (Cao et al., 2004). Therefore, alpine meadows are usually considered to be carbon sinks (Fu et al., 2009), and this capacity may be enhanced under warming conditions (Peng et al., 2014). Our results show that warming resulted in an increase in NEE in the alpine meadow on the Qinghai–Tibetan Plateau in 2012 and 2014. This is consistent with previous results for alpine meadows on the plateau (Hu et al., 2016; Peng et al., 2014; Zhu et al., 2015). However, we found that warming had no significant effect on NEE in 2013. This result may be attributed to delayed vegetation growth in 2013 compared with 2012 and 2014, because of lower spring soil water content in 2013 (Ganjurjav, Gao, Schwartz et al., 2016). Coners et al. (2016) also found that periodic water shortage is likely to influence the alpine meadow which considered as a humid system.

Alpine steppes cover ~30% (ca. 800,000 km^2^) of the Qinghai–Tibetan Plateau, predominantly in the central and western part (Miehe et al., 2011; Ni, 2000). These regions are characterized by lower levels of soil moisture and a drier climate than in the alpine meadow areas. Photosynthesis is more sensitive to soil moisture than is respiration in semiarid steppes (Scott, Hamerlynck, Jenerette, Moran, & Barron‐Gafford, 2010), and consequently, under warming conditions, the NEE may decrease in response to water stress at particularly dry sites or under particularly dry conditions (Jiang et al., 2012; Nagy et al., 2007). Our results confirm that under warming conditions, low soil moisture results in decreased GEP, NEE, and ER.

Cultivated grasslands are important in animal husbandry, especially in regions of low productivity including the Qinghai–Tibetan Plateau (Dong, Long, Hu, Kang, & Pu, 2003; Li & Lin, 2014). Compared with native alpine meadow, in our study, the rates of photosynthesis and respiration of the cultivated grassland increased significantly. Delgado‐Balbuena et al. (2013) also found that NEE increased significantly when the native grassland was converted to cropland; however, carbon loss resulting from exported biomass is the main concern with respect to cultivated grasslands. We found that warming significantly reduced GEP and NEE in the cultivated grassland, but significantly increased SR, which resulted in a net increase in CO_2_ emissions from the soil. Abdalla et al. (2013) also found that under warming conditions, NEE showed a greater degree of variability in cultivated grasslands than in natural grasslands and that this resulted in carbon loss from the cultivated system.

In recent years, the human‐induced land use types were changed significantly on the Qinghai–Tibetan Plateau (Cui & Graf, 2009). Moreover, the changes in land use type have a significant impact on climate in the Qinghai–Tibetan Plateau (Cui, Graf, Langmann, Chen, & Huang, 2006). Reclamation is one of the main land use changes on the Qinghai–Tibetan Plateau. The cultivated grasslands were mainly located in the southern Qinghai–Tibetan plateau and expanded sharply in high altitude regions in past decades (Yang, Shen, & Wang, 2015). The alpine meadow was covered by large area of turf (Kaiser et al., 2008) which can prevent gas emission from soils. After reclamation, the turf layer would be destructed. Then, the soil respiration would increase and carbon uptake would decrease after destruction of turf layer (Babel et al., 2014). Although reclamation could increase productivity, the carbon uptake capacity of cultivated grassland was very sensitive to warming. Decrease in gross carbon uptake and increase in soil respiration resulted in significant decrease in NEE in cultivated grassland under warming.

### 4.3 Uncertainties and implications

The primary uncertainty is about the drawbacks of warming chamber. The open‐top chambers not only increase temperature but also have a significant effect on evapotranspiration and gas exchange due to change in wind. Moreover, the precipitation in the warming chambers is lower than ambient condition due to rain‐sheltering effect, which results in low soil moisture in the chambers. These changes would directly affect C flux in the chambers. Moreover, the chamber method used to measure C flux cannot fully represent the natural C flux because of chamber had changed gas exchange. Nevertheless, above‐mentioned methods are acceptable in view of the fact that warming chamber (Klein, Harte, & Zhao, 2005; Walker et al., 2006) and chamber method measuring C flux (Chivers, Turetsky, Waddington, Harden, & Mcguire, 2009; Jiang et al., 2012; Zhu et al., 2015) have been widely used in grassland ecosystems. At last, we have relatively small database because of we cannot conduct continuous measurement for C flux in chamber warming method. These affect us to draw general conclusion for entire alpine grassland ecosystems on the Qinghai–Tibetan Plateau.

Our results provide insights to understand effects of warming on C flux in different alpine grasslands. We found that water availability is a main regulator of ecosystem C flux under warming, and the NEE decreased in the warming treatments compared with the controls in the alpine steppe and cultivated grasslands. Under future warming and water availability change conditions (Yang et al., 2014), the capacity of the alpine grasslands on the plateau to act as a carbon sink may decline in the Qinghai–Tibetan Plateau (Chen et al., 2013). These results imply that protection of alpine steppe and scientific planning of cultivated grassland should be achieved.

## 5 CONCLUSIONS

The Qinghai–Tibetan Plateau is experiencing significant warming. Our results highlight different responses of C flux to warming in alpine meadow, alpine steppe, and cultivated grassland. Warming induced increase in net C uptake in natural alpine meadow while decrease of that in cultivated grassland and alpine steppe. Cultivated grasslands are very sensitive to warming, and reclamation would increase carbon loss of natural grasslands. Therefore, to adapt to warm and dry climate in the future, overextension of cultivated grassland should be prohibited and scientific planning of cultivated grassland should be achieved.

## CONFLICT OF INTEREST

The authors declare that they have no conflict of interest.

## AUTHORS CONTRIBUTIONS

HG, QG, and YL conceived and designed study; HG, GH, YW, and LD performed research; HG analyzed data; and HG and QG wrote the paper.
