# Supplementary material for: Different responses of ecosystem carbon exchange to warming in three types of alpine grassland on the central Qinghai–Tibetan Plateau
Source: Ecol Evol. 2017 Dec 30;8(3):1507–20. doi: 10.1002/ece3.3741 (PMC5792621; doi:10.1002/ece3.3741)
Supplement: Supplementary file 1 [file ECE3-8-1507-s001.docx]

**Supplementary tables:**

**Table S1** Mean (±SE) aboveground net primary productivity (ANPP), total organic carbon (TOC), total nitrogen (TN) and available nitrogen (AN) at depth of 0-20cm in each treatment in 2014 in alpine meadow, alpine steppe and cultivated grassland sites. The different letters behind the SE value indicated that significant difference between control and warming in the same grassland type.

| Factor | Grassland types | | | | | |
| --- | --- | --- | --- | --- | --- | --- |
|  | Meadow | | Steppe | | Cultivated grassland | |
|  | Control | Warming | Control | Warming | Control | Warming |
| ANPP (g m^-2^) | 37.49±1.04a | 70.90±3.26b | 66.06±3.51a | 54.96±0.54b | 125.92±12.51a | 184.79±5.67b |
| TOC (g kg^-1^) | 34.44±1.57a | 45.48±1.53b | 30.56±1.52a | 26.06±0.45b | 27.21±0.49a | 23.15±0.22b |
| TN (g kg^-1^) | 4.50±0.17a | 6.10±0.17b | 4.08±0.08a | 3.50±0.07b | 3.59±0.08a | 2.82±0.11b |
| AN (mg kg^-1^) | 119.43±21.75a | 155.85±19.40a | 77.36±10.74a | 78.14±7.00a | 113.92±9.77a | 71.15±7.60b |

**Table S2** Linear regression of ecosystem C fluxes with soil temperature (ST), soil water content (SWC) and in alpine meadow, alpine steppe and cultivated grassland in central Qinghai-Tibetan Plateau.

| Ecosystem carbon fluxes | Grassland types | Treatments | Equations | r^2^ | p |
| --- | --- | --- | --- | --- | --- |
| GEP | Meadow | Control | *y* = 0.522*ST* + 46.118*SWC* – 7.737 | 0.209 | <0.001 |
|  |  | Warming | *y* = 0.673*ST* + 48.407*SWC* – 9.775 | 0.328 | <0.001 |
|  | Steppe | Control | *y* = -0.882*ST* + 16.359 | 0.279 | 0.014 |
|  |  | Warming | *y* = -0.703*ST* + 13.085 | 0.413 | <0.001 |
|  | Cultivated grassland | Control | *y* = 0.909*ST* + 69.322*SWC* – 11.350 | 0.259 | <0.001 |
|  |  | Warming | *y* = 125.091*SWC* – 4.693 | 0.182 | <0.001 |
| NEE | Meadow | Control | *y* = 0.488*ST* + 39.584*SWC* – 8.577 | 0.259 | <0.001 |
|  |  | Warming | *y* = 0.545*ST* + 42.987*SWC* – 9.555 | 0.363 | <0.001 |
|  | Steppe | Control | *y* = -0.587*ST* + 10.159 | 0.201 | 0.041 |
|  |  | Warming | *y* = -0.396 *ST* + 6.777 | 0.275 | 0.004 |
|  | Cultivated grassland | Control | *y* = 0.538*ST* + 49.350*SWC* – 7.979 | 0.253 | <0.001 |
|  |  | Warming | *y* = 102.370*SWC* – 7.214 | 0.220 | <0.001 |
| ER | Meadow | Control | *y* = 0.112*ST* + 10.409*SWC* – 0.527 | 0.103 | 0.004 |
|  |  | Warming | *y* = 0.235*ST* – 0.405 | 0.133 | <0.001 |
|  | Steppe | Control | *y* = -0.296*ST* + 6.198 | 0.425 | 0.001 |
|  |  | Warming | *y* = -0.266 *ST* + 5.749 | 0.692 | <0.001 |
|  | Cultivated grassland | Control | *y* = 0.356*ST* + 20.406*SWC* – 2.865 | 0.218 | <0.001 |
|  |  | Warming | *y* = 0.412*ST* + 28.514*SWC* – 3.811 | 0.231 | <0.001 |
| SR | Meadow | Control | *y* = 0.055*ST* + 0.390 | 0.063 | 0.009 |
|  |  | Warming | *y* = 0.117*ST* – 0.056 | 0.137 | <0.001 |
|  | Steppe | Control | —— | —— | —— |
|  |  | Warming | *y* = -0.108 *ST* + 3.023 | 0.518 | <0.001 |
|  | Cultivated grassland | Control | ­­­—— | —— | —— |
|  |  | Warming | *y* = 0.169*ST* + 0.918 | 0.081 | 0.010 |
